# Disrupting prolonged sitting reduces IL-8 and lower leg swell in active young adults

**DOI:** 10.1186/s13102-019-0138-4

**Published:** 2019-10-18

**Authors:** Shilpa Dogra, Mitchell Wolf, Michael P. Jeffrey, Ryan C. A. Foley, Heather Logan-Sprenger, Holly Jones-Taggart, Julia M. Green-Johnson

**Affiliations:** 1 Faculty of Health Sciences (Kinesiology), University of Ontario Institute of Technology, 2000 Simcoe St N, Oshawa, ON L1G-0C5 Canada; 2 Faculty of Science, University of Ontario Institute of Technology, 2000 Simcoe St N, Oshawa, ON L1G-0C5 Canada

**Keywords:** Sedentary time, Inflammation, Exercise, Cytokines

## Abstract

**Background:**

Evidence suggests that disrupting prolonged bouts of sitting with short bouts of physical activity can significantly reduce blood glucose and improve insulin sensitivity; however, limited research is available on the impact of such disruptions on inflammation and swelling. The purpose of this study was to determine whether short bouts of exercise performed each hour during a 4 h sitting session were able to negate the effects of *prolonged sitting* (PS) on several cardiometabolic outcomes.

**Methods:**

Eligible participants (*n* = 10) attended two laboratory sessions: PS (uninterrupted sitting for 4 h) and *disrupted sitting* (DS; 4 h sitting session disrupted by 3 min of exercise each hour (60-s warm-up at 50 W, 5 s of unloaded cycling, 20-s sprint at 5% body weight, and 95-s cool-down at 50 W)). The exercise bouts were performed at minute 60, 120, and 180. Blood and saliva samples, and measures of heart rate and blood pressure were assessed before (T1) and after (T2) each session; leg swell was measured continuously.

**Results:**

Concentrations of salivary IL-8 increased during PS (T1: 0.19 ± 0.32; T2: 0.50 ± 1.00 pg/μg of protein) but decreased during DS (T1: 0.41 ± 0.23; T2: 0.22 ± 0.11 pg/μg of protein, *d:* 0.51, *p* = 0.002). Leg swell increased and plateaued in PS, but was attenuated during DS.

**Conclusion:**

It appears that short bouts of exercise significantly reduce swelling in the lower leg and IL-8 levels in the saliva, indicating that even among healthy, active, young adults, disrupting prolonged sitting can significantly reduce swelling and systemic inflammation.

## Background

Adults spend approximately 10 h of their day engaging in sedentary behaviours [1]. Sedentary behaviour, that is, waking activities performed in a seated or reclined posture that require less than or equal to 1.5 metabolic equivalents, is associated with a diverse range of negative health outcomes [2, 3]. While there is evidence to suggest that engaging in regular physical activity of moderate to vigorous intensity can negate the detrimental effects of sedentary behaviour, few individuals are accumulating the 60–75 min per day that this requires [4]. Thus, feasible strategies are needed to minimize the effects of engaging in high volumes of sedentary time.

Data from laboratory studies provide promising evidence that breaking up prolonged bouts of sitting can significantly and positively influence a range of cardiometabolic outcomes in adults [5]. For example, Peddie et al. [6] showed that regular activity breaks (1 min and 40 s, every 30 min) over a 9 h sitting session led to significant improvements in plasma glucose and plasma triglycerides when compared to either sitting for 9 h, or compared to 30 min of walking prior to sitting for 9 h (*n* = 70). This suggests that breaking up sedentary bouts may be more important for cardiometabolic health than engaging in one bout of moderate to vigorous intensity physical activity in the day. Disrupting prolonged bouts of sitting using similar models has also been shown to improve endothelial function [7], oxidative stress [8] and blood pressure [9].

Chronic low-grade inflammation is implicated in the pathophysiology of several chronic diseases [10] and may be worsened among those who accumulate high volumes of sedentary time [11, 12]. Concentrations of circulating pro-inflammatory cytokines and chemokines such as interleukin (IL)-6, IL-8, IL-1β, and tumor necrosis factor (TNF) α have been shown to be altered in response to exercise interventions [13]. In a study of young sedentary (*n* = 10) and trained men (*n* = 10), 30 min of high intensity treadmill running was found to increase IL-6 and IL-8 significantly in both groups. While there is some research on the response of such cytokines to acute and chronic exercise among in adults [14], little work has been done to determine the impact of disrupting sitting on these inflammatory biomarkers.

Prolonged sitting is also associated with significant edema in the lower leg, and thus increased risk of venous thrombosis [15]. Studies of prolonged sitting during flight simulations [16] and video gaming [17, 18] have shown significant increases in lower leg swelling. Furthermore, adults who sit at work for extended periods have also been shown to be at increased risk of venous thromboembolism [19]. While frequent light activity such as fidgeting is sufficient to negate such edema in the leg [20], little research has looked at the impact of hourly activity on lower leg edema. Hourly activity may be more feasible among those who do not have the predisposition to fidget [21].

The majority of laboratory based studies to date have examined the impact of light to moderate intensities of exercise performed every 20–30 min during prolonged sitting. However, high intensity interval exercise is equal, and in some cases superior, in terms of its impact on health and fitness outcomes to moderate intensity exercise [22]. Thus, the purpose of this research was to determine whether short bouts of high intensity exercise performed each hour during a 4 h sitting session were able to negate the effects of prolonged sitting on cardiovascular and inflammatory measures. Based on previous research, we hypothesized that hourly high intensity exercise would attenuate the inflammatory and cardiovascular response to prolonged sitting.

## Methods

### Study design and experimental protocol

This was an experimental study in which eligible participants attended a baseline and an experimental laboratory session. The baseline session was a *prolonged sitting* session (PS) wherein participants were instructed to sit for 4 h without moving. The experimental session was a *disrupted sitting session* (DS) wherein a 4 h sitting session was broken up with 3 min of exercise. Exercise was performed on a cycle ergometer using the following protocol: 60-swarm-up at 50 W, 5 s of unloaded cycling, 20-s sprint at 5% body weight, and 95-scool-down at 50 W. This protocol has been used effectively in previous research [23]. The exercise bouts were performed at minute 60, 120, and 180 of the 4 h session for a total activity time of 9 min, with a total of 1 min of high intensity exercise. Sessions were scheduled a minimum of 5 days apart to ensure there was no effect of the previous session. In both sessions, participants were permitted to weight shift but were asked to keep their right leg still. They were also allowed to watch television or work on a laptop. Those requiring use of the toilet were wheeled there and back in the chair they were sitting in.

### Participants

Inclusion was limited to adults aged 18–44 years of age who were non-obese (BMI < 30 kg/m^2^) and had no major cardiometabolic, respiratory, or musculoskeletal conditions that would affect their ability to participate in maximal exercise. Participants who had an acute infection, smoked, or used anti-inflammatory medications were excluded.

Participants (*n* = 10, 5 female) arrived to the laboratory fasted (8 h) but were permitted to drink water. Upon completion of resting measures, they were provided a breakfast consisting of a burrito (450 cal, 11 g of protein, 24 g of fat, and 47 g carbohydrate) and 200 mL of orange juice (100 cal, 1 g protein, 23 g carbohydrates). The same amount of juice was provided at 1230 along with a granola bar (110 cal, 1 g of protein, 3 g of fat, 19 g of carbohydrate). Participants were permitted to drink water throughout the 4 h session.

### Measures

The *Physical Activity and Sedentary Behaviour Questionnaire* was used to determine weekly aerobic physical activity levels in mins/week, as well as weekly engagement in strength training, and sitting time in hours/day, to describe the sample. This questionnaire has been shown to be valid for use in adults [24].

Blood and saliva were sampled, and heart rate and blood pressure were assessed prior to commencement of the experiment (T1) and then again at the end of the 4 h session (T2). Stature and mass were assessed using the Detectco medical scale (Detecto, Webb City, MO, USA).

Saliva samples were collected using oral swabs (Salimetrics SalivaBio, Salimetrics LLC, State College, PA, USA) which were placed under the tongue for 4–6 min. Swabs were immediately centrifuged and stored at -80 °C. Total protein content within each saliva sample was determined using the Coomassie Plus Protein Assay Reagent (Thermo Fisher Scientific, MA, USA). Levels of interleukin (IL)-8 (R&D Systems, Catalog# DY208), IL-1β (R&D Systems, Catalog# DY201), IL-6 (R&D Systems, Catalog# DY206), and tumor necrosis factor (TNF) α (R&D Systems, Catalog# DY210) were determined using enzyme-linked immunosorbent assays following manufacturer’s protocols (R&D Systems, MN, USA). Cytokine levels were standardized with the amount of total protein content found within each saliva sample and are expressed as *pg/*μg *of protein*.

A lower leg cuff-style strain gauge was used to assess changes in swell over the 4 h session. Technical details of the strain gauge are described elsewhere [25]. The device was affixed to the leg at the midpoint between the lateral malleolus and the lateral condyle of the fibula. The strain sensing element was positioned on the lateral aspect of the leg for comfort while participants cycled. A ball-chain rope was tightened by the experimenter so the coil spring remained under tension at all times, creating a linear relationship between leg swell and strain gauge output voltage while minimizing non-swell related fluctuations. The strain gauge was calibrated to a sensitivity of 0.2592 V/1 mm of swell, enabling detection of minute changes in leg circumference change. Signals were sampled at 1000 Hz and smoothed using a dual-pass 2nd order Butterworth filter with a 2 Hz cuttoff using MATLAB (v2018b, Mathworks Inc., Natic MA, USA). Baseline activity was removed from the signal to normalize participant data to a steady ‘rest’ value after 30 min of quiet sitting before the experimental collection began [25].

Whole blood samples were collected from a finger prick using a lancet (HTL-STREFA, Inc. Marietta, GA, USA) and capillary tube (Alere San Diego Inc. San Diego, CA, USA). Samples were immediately loaded on to a cassette and analyzed using a point of care system (Alere Cholestech LDX Analyzer, San Diego, CA, USA). The analyzer provided data on total cholesterol (mmol/L), high-density lipoproteins (mmol/L), triglycerides (mmol/L), low-density lipoproteins (mmol/L), and glucose (mmol/L).

Heart rate and blood pressure were assessed using an automatic sphygmomanometer (A&D Medical Digital Blood Pressure Monitor, Model UA -767FAM, A&D Engineering, Inc. San Jose, CA, USA).

All protocols were approved by the University of Ontario Institute of Technology Research Ethics Board and Biosafety Committee. All participants provided written consent prior to participation.

### Statistical analysis

Means and standard deviations were calculated for baseline characteristics as well as data from T1 and T2 from PS and DS.

Analysis of covariance was used to detect differences between PS and DS at T2; T1 was used as a covariate to account for baseline differences between sessions. Partial eta squared was used to estimate effect sizes for sessions (small: 0.01, medium: 0.09, large: 0.25). Repeated measures analysis of variance was used to determine differences in leg swell between PS and DS and multiple time points corresponding to the time of the exercise bouts during DS. Pairwise comparisons with a bonferroni correction applied were conducted to detect where the differences were. All statistics were conducted in SPSS (v25, IBM).

Our primary outcome of interest was inflammatory markers (IL-8, IL-6, TNF-α, and IL-1β). Effect sizes for these inflammatory markers in this experimental design are not available. Therefore, based on a study by de Souza et al. [26] where the effects of a high intensity exercise session on a number of cytokines was assessed, we aimed to recruit a sample of 10 adults. This sample was also sufficient to detect differences in blood pressure, as per previous research [27, 28].

## Results

The sample was young (24.7 ± 2.9 years), had a normal BMI (23.0 ± 2.1 mm/kg^2^), was aerobically active (228.0 ± 136.1 min/wk), engaged in strength training (2.7 ± 1.4 sessions/week) and a large volume of sedentary time (7.3 ± 1.26 h per day).

Changes in PS and DS as well as corresponding effect sizes are available in Table 1. For HR, there was a significant main effect for session (*p* < 0.01) indicating a significantly higher HR at the end of DS compared to PS. No significant differences were observed for SBP or DBP. For blood glucose, there was a significant main effect for session (*p* = 0.034) indicating a higher blood glucose at the end of DS compared to PS. No significant differences were observed for any of the other blood markers.

**Table 1:** Changes in measures during prolonged sitting (PS, *n* = 10) and disrupted sitting (DS, *n* = 10) sessions

	Prolonged Sitting		Disrupted Sitting		Partial Eta Squared
	T1	T2	T1	T2	
Heart Rate (bpm)	62.5 ± 12.0	63.8 ± 13.0	63.3 ± 11.0	73.3 ± 9.1*	0.36
Systolic Blood Pressure (mmHg)	117.2 ± 11.9	110.6 ± 15.0	111.4 ± 12.5	110.5 ± 10.7	0.09
Diastolic Blood Pressure (mmHg)	74.2 ± 6.9	71.6 ± 6.4	71.4 ± 6.0	70.0 ± 7.8	0.10
Total Cholesterol (mmol/L)	4.4 ± 0.8	4.6 ± 0.8	4.3 ± 0.5	4.4 ± 0.7	0.10
HDL Cholesterol (mmol/L)	1.4 ± 0.4	1.3 ± 0.4	1.3 ± 0.3	1.3 ± 0.3	0.00
Triglycerides (mmol/L)	1.2 ± 0.5	1.6 ± 0.9	1.0 ± 0.3	1.4 ± 0.5	0.01
LDL Cholesterol (mmol/L)	2.5 ± 1.1	2.5 ± 0.6	2.5 ± 0.7	2.4 ± 0.7	0.04
Glucose (mmol/L)	4.5 ± 0.5	5.0 ± 0.7	4.7 ± 0.3	5.7 ± 0.8*	0.24
IL-1β (pg/μg of protein)	0.09 ± 0.01	0.01 ± 0.02	0.07 ± 0.09	0.01 ± 0.02	0.00

Partial eta squared: small (0.01), medium (0.09), and large (0.25)

*p < 0.05; means ± SD

**Fig. 1:**
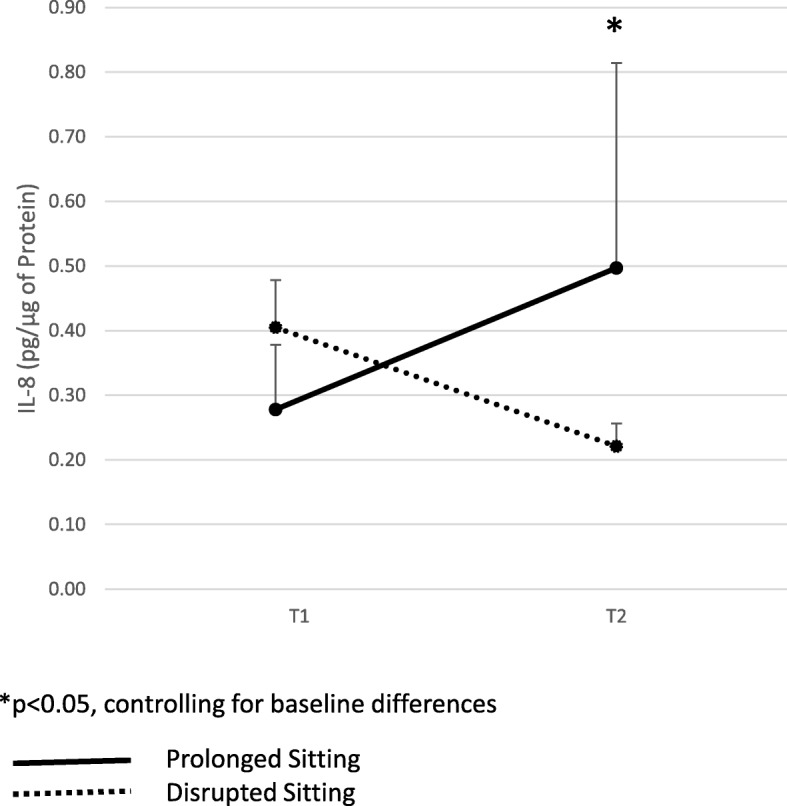
Interleukin-8 Responses from Prolonged (PS, *n* = 10) and Disrupted (DS, *n* = 10) Sitting Sessions (means ± standard error)

Concentrations of TNF-α and IL-6 within the saliva samples were below the limit of quantification (TNF-α: 15.6 pg/mL and IL-6: 9.38 pg/mL), while IL-1β was not significantly different between PS and DS. A significant interaction for session by baseline IL-8 (*p* < 0.001) was detected, indicating that salivary IL-8 concentrations increased during PS (T1: 0.19 ± 0.32, T2: 0.50 ± 1.00) but decreased in DS (T1: 0.41 ± 0.23, T2: 0.22 ± 0.11, partial eta squared: 0.24) (Fig. 1).

**Fig. 2:**
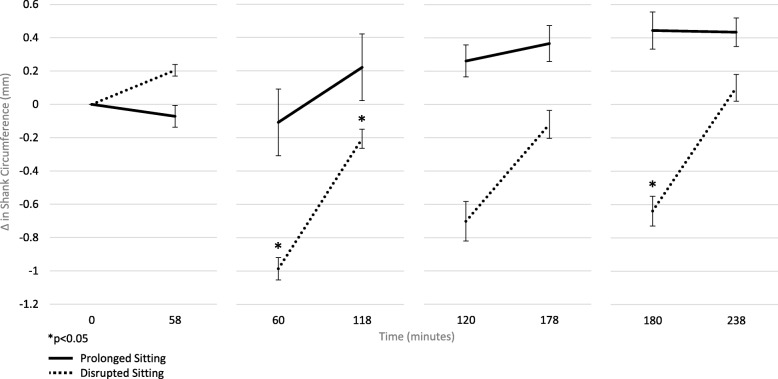
Leg Swell Response from Prolonged (PS, *n* = 10) and Disrupted (DS, *n* = 10) Sitting Sessions (means ± standard error)

Changes in leg swell (PS: Δ 0.39 ± 0.97; DS: Δ -0.09 ± 0.95) are depicted in Fig. 2. There was a significant interaction (*p* = 0.001) suggesting differences between PS and DS at time-points between T1 and T2. There were significant differences between groups at minute 60 (PS: − 0.11 ± 0.70, DS: − 0.99 ± 0.67 mm, *p* = 0.01) and at minute 180 (PS: 0.44 ± 1.11, DS:-0.64 ± 0.90 mm, *p* = 0.02), and a trend for significance at minute 120 (PS: 0.26 ± 0.96, DS: − 0.70 ± 1.18 mm, *p* = 0.06), that is, swelling was significantly reduced immediately after an exercise bout compared to prolonged sitting. Leg swell was also significantly lower at minute 118 (PS: 0.22 ± 0.90, DS: − 0.21 ± 0.58 mm, *p* < 0.01), that is, swelling remained significantly lower after sitting for an hour.

## Discussion

We sought to determine whether high intensity exercise would attenuate the pro-inflammatory and cardiovascular response to 4 h of prolonged sitting. Our primary finding is that sitting disrupted by a 3 min bout of exercise reduced swelling in the lower leg and reduced IL-8 among healthy, active, and young adults. These findings are the first, to our knowledge, to assess the impact of high intensity exercise on prolonged sitting, and provide promising data to support breaking up prolonged sitting, even among those who are young, active, and healthy. These findings have important implications for the methodological design of future research studies assessing the impact of sedentary time on chronic, systemic inflammation.

In our study, there was a trend for an increase and then a plateau in leg swelling over the 4 h prolonged sitting period. This is in contrast to research on older healthy men that showed an exponential increase in leg swelling in the first 45 min of sitting followed by a linear increase in the subsequent 105 min of sitting, with no evidence of a plateau [29]. These differences may be due to the age and activity levels of the sample. We also found that short bouts of exercise led to a decrease in lower leg swell immediately after exercise when compared to prolonged sitting. Importantly, the gradual increase observed in leg swell during the 60 min of sitting after an exercise bout remained attenuated when compared to prolonged sitting. These findings are in line with previous research. In a study of healthy young men (*n* = 13), intermittent movement including light walking, knee bends, and heel-raises during standing work were found to significantly reduce leg swell and associated subjective complaints [30]. Similarly, a study assessing the effect of light intensity activity breaks on endothelial function of the superficial femoral artery during a 3 h sitting session found that activity breaks were able to attenuate the decline in endothelial function [7]. Together, these studies indicate that disrupting prolonged sitting may be critical for vascular health. Our study is the first to provide promising evidence that hourly bouts of exercise can significantly reduce the risks associated with lower leg swelling [31] even among active young adults, that is, among those without any known impairment in cardiovascular function. The magnitude of effect *may be* even greater among those with impaired cardiovascular function.

With regard to salivary cytokines, we found a significant difference in IL-8 concentrations. IL-8 concentrations increased during prolonged sitting, but decreased after exercise, indicating a decrease in this biomarker of inflammation within 1 h of completing the final exercise bout. IL-8 is a pro-inflammatory chemokine [13] associated with chronic inflammation [32]; a systemic increase in IL-8 is typically observed in response to intense exercise [33, 34]. In a study of healthy lean (*n* = 10) and overweight (*n* = 12) men who performed a high intensity interval or moderate intensity interval exercise session, IL-8 levels were found to increase significantly immediately after exercise but decrease at 30 min post-exercise [26]. Although we did not test cytokine levels immediately post-exercise, the decrease observed 1 h after the last exercise bout is in line with these findings, and suggest an impact of this intervention on a key mediator of low grade systemic inflammation.

Concentrations of IL-1β remained unchanged, while IL-6 and TNFα were undetectable; this may reflect the nature of the intervention, as impact on these pro-inflammatory cytokines varies greatly with exercise duration and intensity [13]. Recent studies indicate that levels of salivary cytokines can be correlated to those found in serum in some disease models [35]. It is worth emphasizing again that our sample was young, active and healthy, thus, results may be of even greater magnitude among those at risk or with existing chronic conditions associated with low-grade systemic inflammation. It is also important to note that we looked at cytokines in saliva, not blood. Limited research exists on this non-invasive method of assessment; however, from data available to date, saliva may be a valid tool to assess acute responses to stressors such as exercise [14]. Thus, future research is needed to better characterize normal values using saliva samples.

The responses observed in cardiovascular measures were also somewhat novel. Research to date indicates that prolonged sitting is associated with an increase in blood pressure, blood glucose, triglycerides, and other cardiometabolic risk factors [6, 28, 36, 37]; however, in our sample, no significant changes were observed. This is likely due to the difference in samples. We used a young, active, and healthy group of adults, while previous research has focused on sedentary office workers. Of note, we did see significant differences in heart rate and blood glucose when comparing prolonged sitting to disrupted sitting such that heart rate and blood glucose levels were higher at the end of the disrupted sitting session, indicating that participants had not yet returned to baseline 60 min after the last exercise bout.

There are some weaknesses that should be considered in interpreting our findings. First, we did not control for exercise performed the day before participants completed the two sessions. This may have influenced resting measures as well as responses to the sessions; however, it is important to note that we had a highly active sample, thus most participants likely were active the day before. Second, this young, active, and healthy sample was homogenous, limiting generalizability to older samples or adults with chronic conditions; however, the data provide conservative estimates of potential treatment effects in these higher risk groups. Third, we did not record water intake during the session. This may have influenced leg swell, and should be considered in future research. Fourth, little research to date has examined sex-differences in the response to prolonged and disrupted sitting. Research indicates that menstrual cycles may influence the basal levels of salivary cytokines [38]. While we were underpowered to perform sex-based analyses, we did include an equal number of males and females in our study. Fifth, while we used a cycle ergometer, a piece of equipment that may not be readily available in the workplace, recent research suggests that the benefits of “exercise snacking” [39, 40], that is, short bursts of higher intensity exercise, can be obtained by running up the stairs. Thus, this type of intervention may be feasible in school and work settings. Finally, we did not employ a randomized-crossover design due to scheduling logistics; this design should be used in future research in order to limit minimize potential order effects. Future research should also use serial measures of cytokines to determine the changes over the course of a 4 h session.

## Conclusion

It appears that short bouts of exercise performed at high intensity are able to significantly reduce swelling in the lower leg and IL-8 levels in the saliva indicating that even among healthy and active young adults, disrupting prolonged sitting can significantly reduce swelling and systemic inflammation. Future research is needed using older adults or those at high risk of non-communicable disease to determine whether such disruption can provide similar benefit.

## Data Availability

The datasets used and/or analysed during the current study are available from the corresponding author on reasonable request.

## Abbreviations

BMI: Body Mass Index
DS: Disrupted sitting
IL: Interleukin
PS: Prolonged sitting
T1: Time 1 (pre-experiment)
T2: Time 2 (post-experiment)
TNF: Tumor necrosis factor
